# Supplementary material for: Chromothripsis during telomere crisis is independent of NHEJ, and consistent with a replicative origin
Source: Genome Res. 2019 May;29(5):737–49. doi: 10.1101/gr.240705.118 (PMC6499312; doi:10.1101/gr.240705.118)
Supplement: Supplemental Material [file supp_gr.240705.118_Supplemental_file_1.zip › contigs/annotated_contigs/DB41/contig.4.DB41_length_724_mean_cov_12.8743093923.docx]

**DB41_length_724_mean_cov_12.8743093923**

```
ATGTTCTCATTCTCGAGGGGCCCCTGCTCCCCCCATCCCTCCCCAGCCACATGGCTGGACTGAAGCTTGCACCTGTTTTGATATCCAGG
 >chr17:75870231-75870476 + E=1e-135
GAAGGGTCTAGCCCAGTAGGTCACCTGTCTGATGGCTGCCCCTTAAATGCAGGTGGCATGCCCAGAAAGACTCAACCTGCCTCTGAAAC

CCTCCCTGGTCCCCACAGAGGGACAGCCACCCCCCTCCCTCCCTGCACCATCACCAAACACAGATC|A|AATGGTAATAATCATGAAAT
                                                                   >chr12:39237837-3923
GTATAATGTTATTATAAAATATTTTAGATGGATCTCAACACTTTGAGGAAAAATAAGTTGTTTTATTTCTTCTAGTAGTTGTCCCAACA
7963 - E=5e-64
CCATGTCAACTGTGGT|CATGCTGTTCCTTCAACGAACTACATTACTACAGTGAAAATTGATCACACAAAATGAGGTCATTTTTGTCAC
                  >chr12:39238735-39238821 + E=6e-40
ACCCAACTAA|AACA|AACATCCCTACAACAAAAACTCAAGATGAAAGAGCCAGAGAGAAGCAAATTAAAGGCTTGGAAAGATGCAAAT
           >chr2:16239909-16240181 + E=8e-152
CAAGTCTTTGTCTAGAGTAGTGGAGCCAAGAACCTGGTGGCTTTACTCAACAGGTATTCACTGAGCACCTACTCTGTGTAGGAGACCCT

GAGAGATGATAACCTCTCAGTCTTTTTTCTTTTTTCCTTTAAATAGGGATGGGTTCTCGCTATGTTAACCAGGCTGGACTTGAACTCCT

GGACTCAAGTGATTCTT
```
